# ^17^O-labeled water distribution in the human inner ear: Insights into lymphatic dynamics and vestibular function

**DOI:** 10.3389/fneur.2022.1016577

**Published:** 2022-11-03

**Authors:** Tadao Yoshida, Shinji Naganawa, Masumi Kobayashi, Satofumi Sugimoto, Naomi Katayama, Tsutomu Nakashima, Yutaka Kato, Kazushige Ichikawa, Hiroshi Yamaguchi, Kazuki Nishida, Michihiko Sone

**Affiliations:** ^1^Department of Otorhinolaryngology, Nagoya University Graduate School of Medicine, Nagoya, Japan; ^2^Department of Radiology, Nagoya University Graduate School of Medicine, Nagoya, Japan; ^3^Department of Health and Nutrition, Faculty of Health and Sciences, Nagoya Women's University, Nagoya, Japan; ^4^Department of Rehabilitation, Ichinomiya Medical Treatment and Habilitation Center, Ichinomiya, Japan; ^5^Department of Radiological Technology, Nagoya University Hospital, Nagoya, Japan; ^6^Medical Branch of Radioisotope Research Center, Nagoya University, Nagoya, Japan; ^7^Department of Biostatistics Section, Center for Advanced Medicine and Clinical Research, Nagoya University Graduate School of Medicine, Nagoya, Japan

**Keywords:** ^17^O-labeled water, MRI, perilymph, endolymph, vertigo

## Abstract

We evaluated the inner ear distribution of ^17^O-labeled saline administered to the human tympanic cavity. Magnetic resonance imaging was performed after intratympanic administration in five healthy volunteers and one patient with cochlear endolymphatic hydrops. In all volunteers, ^17^O-labeled water permeated the cochlear basal turn and vestibule at 30 min and disappeared gradually within 2–4 h. All participants experienced positional vertigo lasting a few hours to a few days. Visualization of ^17^O-labeled water distribution in the endolymphatic space of the posterior ampulla showed indistinct separation of endolymph and perilymph in the cochlea and most of the vestibule in all participants. Intralabyrinthine distribution of ^17^O-labeled water differed from that in previous reports of intratympanically administered gadolinium-based contrast agent. ^17^O-labeled water in the endolymphatic space may cause heavier endolymph and positional vertigo. These results of this study may add new insights for investigating the distribution and the effects of molecules in the inner ear after the intratympanic administration in living humans.

## Introduction

^17^O has a low natural abundance of 0.038% and is the only stable isotope of oxygen that produces signal changes in proton magnetic resonance imaging (MRI) (1–3). Because of its low natural abundance, enrichment is required for the detection of ^17^O (2). The direct detection method of ^17^O uses the Larmor frequency of the ^17^O nucleus in humans and animals (4–6), and requires specific hardware. In the indirect detection method, the proton of water molecules labeled with ^17^O can be viewed as a signal change because of T2 shortening (1, 3, 7). Given that ^17^O-labeled water is a water molecule, it can be safely administered without inducing an allergic reaction in living organisms or risk in patients with renal failure. The safety of intravenous and intrathecal administration of ^17^O-labeled water has been reported in humans (3, 8).

^15^O-positron emission tomography (PET) is considered to be the gold standard for the quantitative analysis of cerebral blood flow and oxygen metabolism. However, ^15^O has a short half-life of 122 s, requires a cyclotron for quantification, and involves radiation exposure (9). The spatial resolution of PET is insufficient for the analysis of tiny structures such as the inner ear. One significant advantage of ^17^O is that it has no half-life as a nuclide, which allows for extended time tracer analysis, and no radiation exposure.

The first visualization of endolymphatic hydrops in a patient with Ménière's disease was achieved using three-dimensional (3-D) fluid-attenuated inversion recovery images at 3 T 24 h after intratympanic administration of a gadolinium-based contrast agent (GBCA), and this method has been used by many researchers since then (10–13). The preferential distribution of GBCA in the perilymph allows endolymphatic hydrops to be imaged (14, 15). Water has a minimal molecular weight (18 for regular water, 19 for ^17^O-labeled water) compared with GBCA (500–800 depending on the type of agent).

In this study, we hypothesized that ^17^O-labeled water administered into the tympanic cavity would penetrate the perilymph through the round window faster than GBCA. The purpose of this study was to evaluate the permeability and distribution of intratympanically administered ^17^O-labeled water in healthy people without auditory vestibular symptoms and in a patient with endolymphatic hydrops.

## Methods

### Participants

Volunteers with no previous auditory or vestibular symptoms were recruited between November 2021 and December 2021. Eligible participants had to be at least 20 years of age; four male volunteers and one female volunteer (mean age: 34 years; age range: 30–42 years) were included. A male patient aged in his 40 s with endolymphatic hydrops in the cochlea noted by previous gadolinium-contrast MRI was also included. The patient had a previous history of acute low-tone sensorineural hearing loss but had no history of vertigo and was currently asymptomatic. The study was approved by the institutional review board of the study institution (No. 2021-0309), and written informed consent was obtained from all participants before enrolment.

^17^O-labeled saline (10 mol% H${}_{2}^{17}$O; Taiyo Nippon Sanso Corp., Tokyo, Japan) was injected intratympanically using a 23-G needle and 1-mL syringe into the left ear of the five volunteers and into the right ear of the patient with endolymphatic hydrops. Each participant was placed in the supine position with their head turned ~30° away from the sagittal line toward the non-injected side ear. The solution was warmed to body temperature before injection. ^17^O-labeled saline was injected until a backflow of fluid into the external ear was observed through a microscope, and the injected volume was 0.6–0.8 mL per participant. After the injection, the participants remained in the supine position for 30 min with the head turned ~60° away from the sagittal line toward the non-injected side ear. This water tracer was made under good manufacturing practice standards for intravenous injection in human participants. In a previous study, 1 mL/kg of 20 mol% H${}_{2}^{17}$O was administered intravenously at a rate of 3 mL/s to 14 volunteers without any adverse effects (3). To our knowledge, the present study is the first to apply this tracer intratympanically in humans.

### MRI

The estimated GBCA concentration in perilymph 24 h after intratympanic administration of GBCA is ~1/625 of that of the administered solution (15). The molecular weight of gadoteridol used for intratympanic administration is 558.7, and that of ^17^O-labeled water is only 19. Therefore, <625 times dilution is expected for ^17^O-labeled water compared with GBCA in the perilymph after intratympanic administration.

### Phantom experiments and sequence optimization in human volunteers

**Figure 1:**
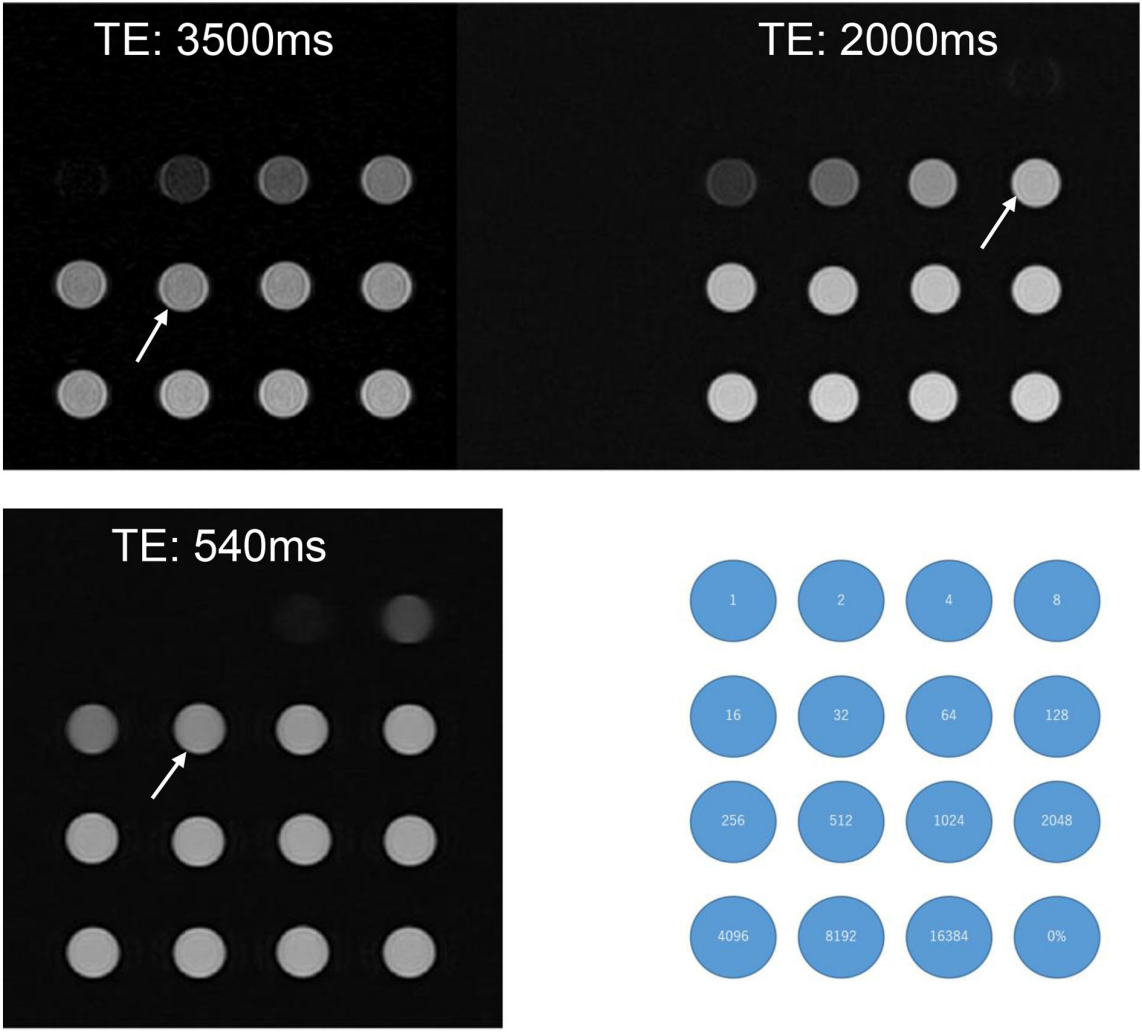
A phantom experiment with test tubes of various ^17^O-saline concentrations. 10 mol% ^17^O-saline was diluted with normal saline. Numbers in the blue circles indicate the dilutions. The right lower corner tube (0%) indicates the tube filled with normal saline. Compared with normal saline, the slight signal decrease for ^17^O-saline was barely recognizable for the 512 times dilution for a TE of 3,500 ms (arrow), 128 times dilution for a TE of 2,000 ms (arrow), and 32 times dilution for a TE of 540 ms (arrow). Even at a TE of 540 ms, dilutions of <8 did not produce a visible signal.

In a pilot study, we performed phantom experiments to optimize the pulse sequence parameters for the scan after intratympanic administration of ^17^O-labeled saline. ^17^O-labeled water shortens the T2 time as an indirect effect in proton MRI (3). We prepared test tubes with various dilutions of 10 mol% ^17^O-labeled saline (Taiyo-Nissan Co. Ltd, Tokyo, Japan) as follows: original solution; dilutions of 2, 4, 8, 16, 32, 64, 128, 192, 256, 512, 1,024, 2,048, 4,096, 8,192, and 16,384 times; and normal saline. To visualize low concentrations of ^17^O-labeled water using T2-weighted images in the human inner ear with fine anatomy, we used hT2W MR cisternography and 3-D turbo spin–echo (SPACE: sampling perfection with application-optimized contrasts using different flip angle evolution) with TEs of 500–5,600 ms (16). In the phantom experiments, we could differentiate the 512 times dilution and normal saline using a TE of 3,500 ms (Figure 1). With an increasing TE, the signal–intensity ratio (SIR) (normal saline/512-times diluted ^17^O-labeled water) increased to 1.00 at a TE of 500 ms, 1.06 at a TE of 2,000 ms, 1.10 at a TE of 3,000 ms, and 1.12 at a TE of 3,500 ms. However, with an increasing TE, the image noise increased. We needed to identify the maximum TE value to visualize the fine inner ear anatomy with a reasonable scan time in humans.

**Figure 2:**
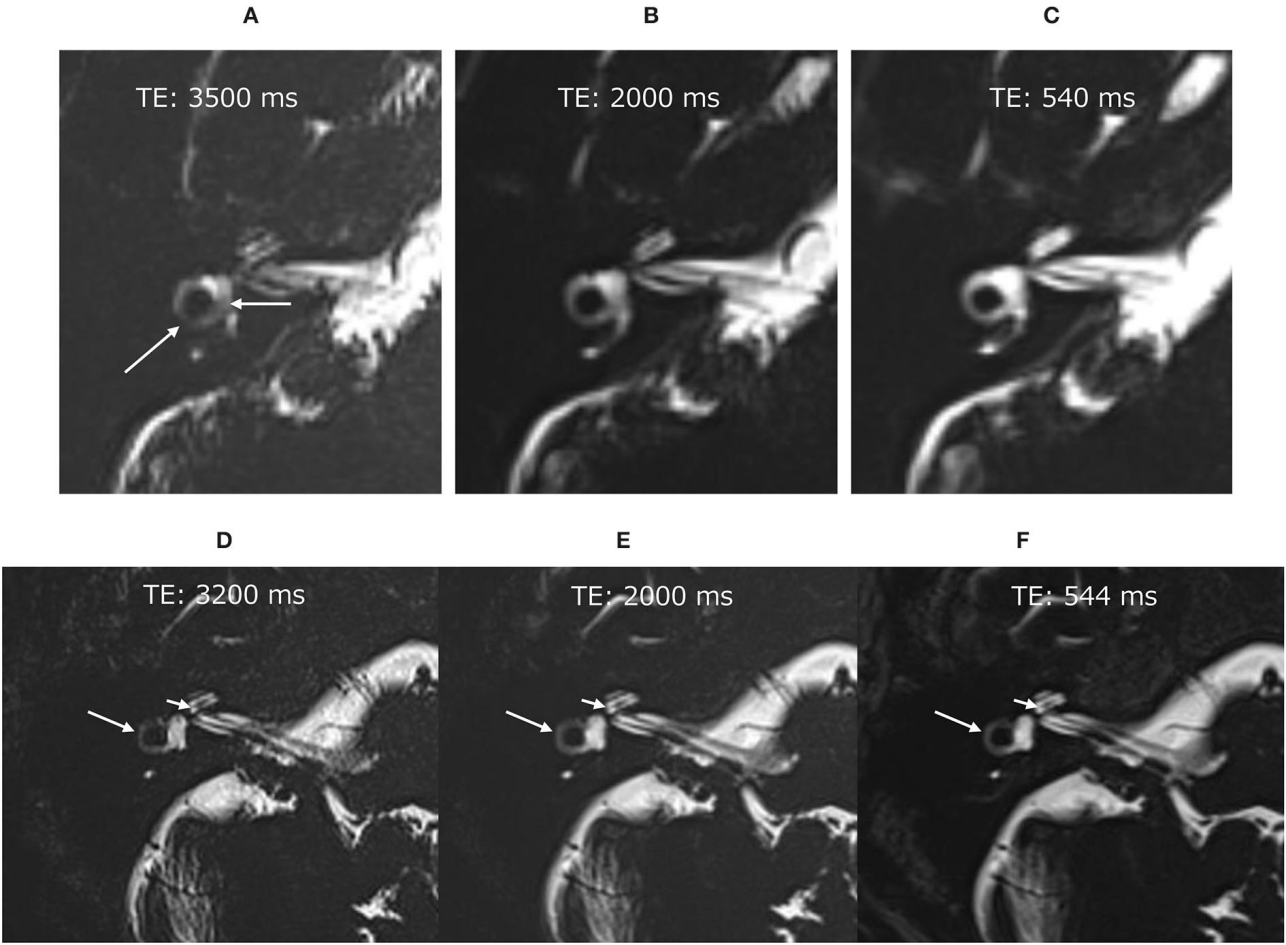
Pilot scans in volunteers without administration of the ^17^O-labeled water tracer. In the image with an echo time (TE) of 3,500 ms **(A)**, the vestibular signal was not uniform, and the lateral semicircular canal was not continuous (arrows). Note that these structures were visualized clearly in the images with a TE of 2,000 ms **(B)** and TE of 540 ms **(C)**. Pilot scans in another volunteer without ^17^O-labeled water tracer administration. Although the image with a TE of 3,200 ms was slightly noisy **(D)**, the anatomy of the labyrinth could be seen clearly. The signal for the vestibule was more uniform in the image with a TE of 3,200 ms **(D)** than in that with a TE of 3,500 ms **(A)**. For example, the osseous spiral lamina of the cochlea (short arrow) and the lateral semicircular canal (arrow) could be identified in all images of this volunteer **(D–F)**.

Before the administration of ^17^O-labeled saline, we scanned three volunteers. The maximum TE to visualize the inner ear anatomy steadily in a reasonable scan time of 6 min was 3,200 ms (Figure 2). We could not predict the exact dilution ratio of ^17^O-labeled water saline in the perilymph nor how fast the ^17^O-labeled water or saline would permeate and distribute within the labyrinth. We decided to use three TEs to accommodate the various concentrations, 540, 2,000, and 3,200 ms, and to obtain images at multiple times. Using multiple TEs and images with various sensitivity to low concentrations of ^17^O-labeled water allowed us to perform image processing of the different TEs and to produce easily recognized images for the visualization of the ^17^O-labeled water distribution. All MRI scans were performed using a 3 T unit (Skyra, Siemens Healthineers, Erlangen, Germany) using a 32-channel array head coil. Details of the scan parameters are shown in Table 1.

**Table 1:** Scan paremeters for 3 kinds of MR cisternography.

**Sequence name**	**Type**	**Repetition time (ms)**	**Echo time (ms)**	**Flip angle (degree)**	**Section thickness/re construction step (mm)**	**Pixel size (mm)**	**Number of slices**	**Echo spacing (ms)**	**Echo train length**	**Echo train duration (ms)**	**Phase partial fourier (%)**	**Field of view (mm)**	**Matrix size**	**Number of excitations**	**GRAPPA**	**Bandwidth (Hz/pixel)**	**Slice oversampling (%)**	**Scan time (min:s)**
MR cisternography 1	3D T2 SPACE	15,000	3,200	90/ constant160	2.0/1.0	0.5×0.5	80	7.23	599	4,345	77	1 65 × 196	324 × 384	1.4	3	434	100	6:00
MR cisternography 2	3D T2 SPACE	15,000	2,000	90/ constant160	2.0/1.0	0.5×0.5	80	7.23	599	4,157	96	165 × 196	324 × 384	1.4	3	434	100	6:00
MR cisternography 3	3D T2 SPACE	4,000	540	90/ constant130	2.0/1.0	0.5×0.5	80	6.11	272	1,369	82	165 × 196	324 × 384	1.4	3	434	100	3:28

SPACE, Sampling perfection with application-optimized contrasts using different flip angle evolutions.

GRAPPA, GeneRalized autocalibrating partial parallel acquisition.

All three sequences utilize a restore magnetization pulse and slab selective excitation pulse.

3D slab is set in an axial orientation.

### MRI scanning

MRI was performed 30 min, 2 h, 4 h, and 24 h after the intratympanic administration of ^17^O-labeled saline in all volunteers. All volunteers underwent hT2W MR cisternography with three different TEs (544, 2,000, and 3,200 ms) for 3 min for a TE of 544 ms and for 6 min for the other TEs. The first scan was followed by an additional imaging using a TE of 3,200 ms 15 min after the start of the first phase in three volunteers. In the male patient with endolymphatic hydrops in the cochlea, the 6 min scan for a TE of 3,200 ms was performed five times and a subsequent 3 min scan for a TE of 540 ms was obtained.

### Evaluation of ^17^O-labeled water contrast effects

The contrast effects on the cochlear and vestibular fluid were evaluated semiquantitatively, as reported previously in patients with sudden deafness or Ménière's disease (17, 18). The SIR was measured three times, and the average SIR value was calculated. The signal intensities of each inner ear were quantified as follows. The measurement sites were the basal and apical–middle turns of the cochlea, vestibular cavity, and ampulla of the posterior semicircular canal.

For the basal turn region of interest (ROI), the slice was selected at least three slices below the center slice on which the cochlear modiolus appeared largest. For the apical–middle turn ROI, the slice was selected at the center slice on which the cochlear modiolus appeared largest. The ROI for the vestibule was drawn on the lowest slice on which the lateral semicircular canal ring was visualized at more than 240° and excluded the semicircular canal and ampulla when the ROI for the vestibule was drawn on MR images with a TE of 540 ms. For the ampulla of the posterior semicircular canal ROI, one slice below the slice on which the vestibule was assessed was selected.

**Figure 3:**
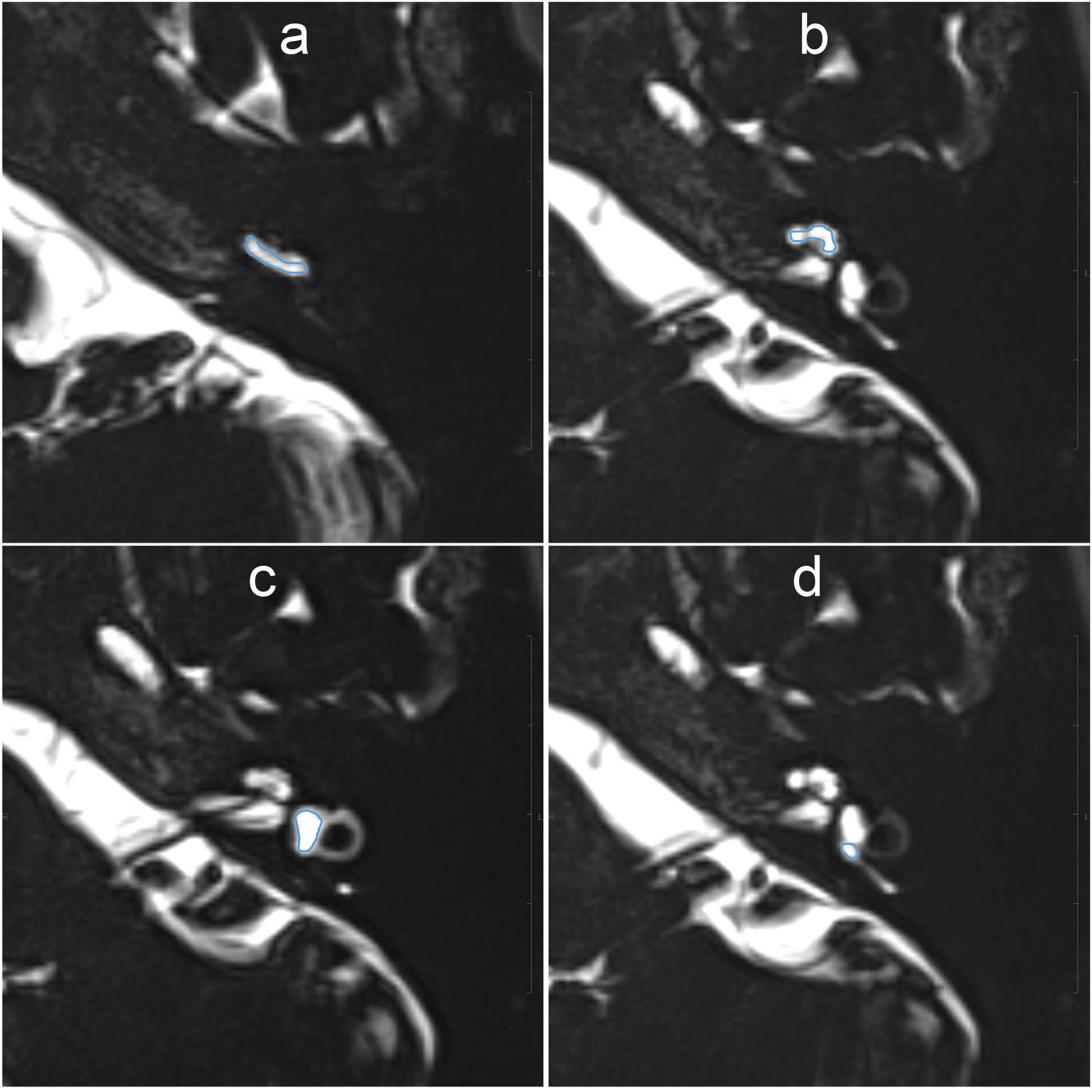
Example images for setting of the region of interest (ROI) on MR images with an echo time (TE) of 540 ms. The scala tympani of the basal turn of the cochlea **(a)**, whole apical–middle turn **(b)**, vestibule **(c)**, and the ampulla of the posterior semicircular canal **(d)** appeared contoured. The ROIs were then copied onto the other TE images. Each signal–intensity ratio was calculated using the signal value of the contralateral inner ear as a control.

For quantitative evaluation, the SIR between the signals of the injected left side and the non-injected right side was measured on MR images with a TE of 3,200 ms. The ROIs were drawn manually on the MR images with a TE of 540 ms, and were copied and pasted onto MR images with a TE of 3,200 ms and onto MR images taken during another time phase (Figure 3). If motion was detected between the scans, fine adjustments were made manually. Using the signal value of the ROI for the right side as a control, each SIR was calculated as the signal intensity of the ROI in the left side divided by that in the right ear. The ROIs were placed using a PACS viewer (Rapid-eye Core, Canon Medical Systems, Tochigi, Japan) by a single observer with 15 years of experience in the evaluation of inner ear MRI.

### Evaluation of positional nystagmus

Nystagmus was evaluated by VOG using a yVOG glass device (Daiichi Medical Company, Tokyo, Japan). VOG evaluates all the three dimensions of eye movements, namely, the horizontal, vertical, and torsional components. Spontaneous nystagmus in the dark was recorded in the left inferior, supine, and right inferior head positions.

## Results

All volunteers received intratympanic administration of ^17^O-labeled saline to their left ear and completed the study protocol. The volunteers had no symptoms of positional vertigo during the 30-min rest period after administration of ^17^O-labeled saline and before the start of the MRI study. However, all volunteers experienced positional vertigo after 30 min following the intratympanic administration of ^17^O-labeled saline lasting 40 min to 6 h. The duration of vertigo for each volunteer was as follows: volunteer #1 for 6 h, volunteer #2 for 4 h, volunteer #3 for 3.5 h, volunteer #4 for 5.5 h, and volunteer #5 for 40 min. We could not record the nystagmus of the volunteers, but observation under Frenzel goggles revealed a horizontal rotatory direction-changing positional geotropic nystagmus. Vertigo and nystagmus lessened when the head position was returned to rest with the injected side up. After the vertigo symptoms disappeared, there were no complaints of auditory vestibular symptoms, and all volunteers returned to their pretreatment state.

**Figure 4:**
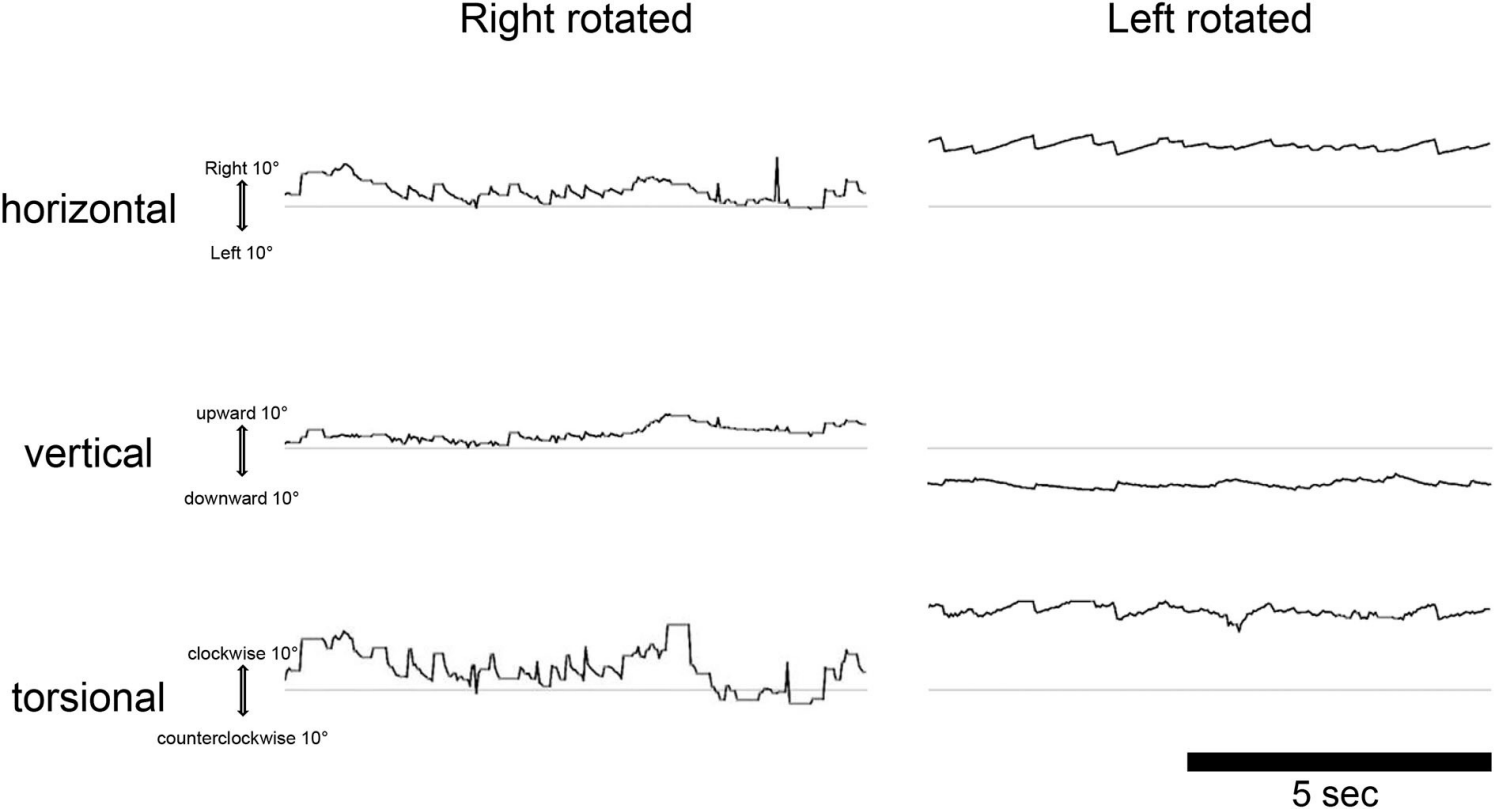
Video oculography findings in the dark 270 min after injection of ^17^O-labeled water in the right (affected) tympanic space in a patient with cochlear endolymphatic hydrops. The eye movements are displayed from top to bottom, as horizontal, vertical, and torsional as indicated. The upward direction of each trace indicates right, up, and clockwise (from the subject view). The ordinate and abscissa indicate the eye position in degrees and time in seconds, respectively. The average slow phase speed of nystagmus was 4°/s to the right, 2°/s down, and 6°/s clockwise for the left rotated, and 8°/s to the left, 3°/s down, and 19°/s counterclockwise for the right rotated.

**Figure 5:**
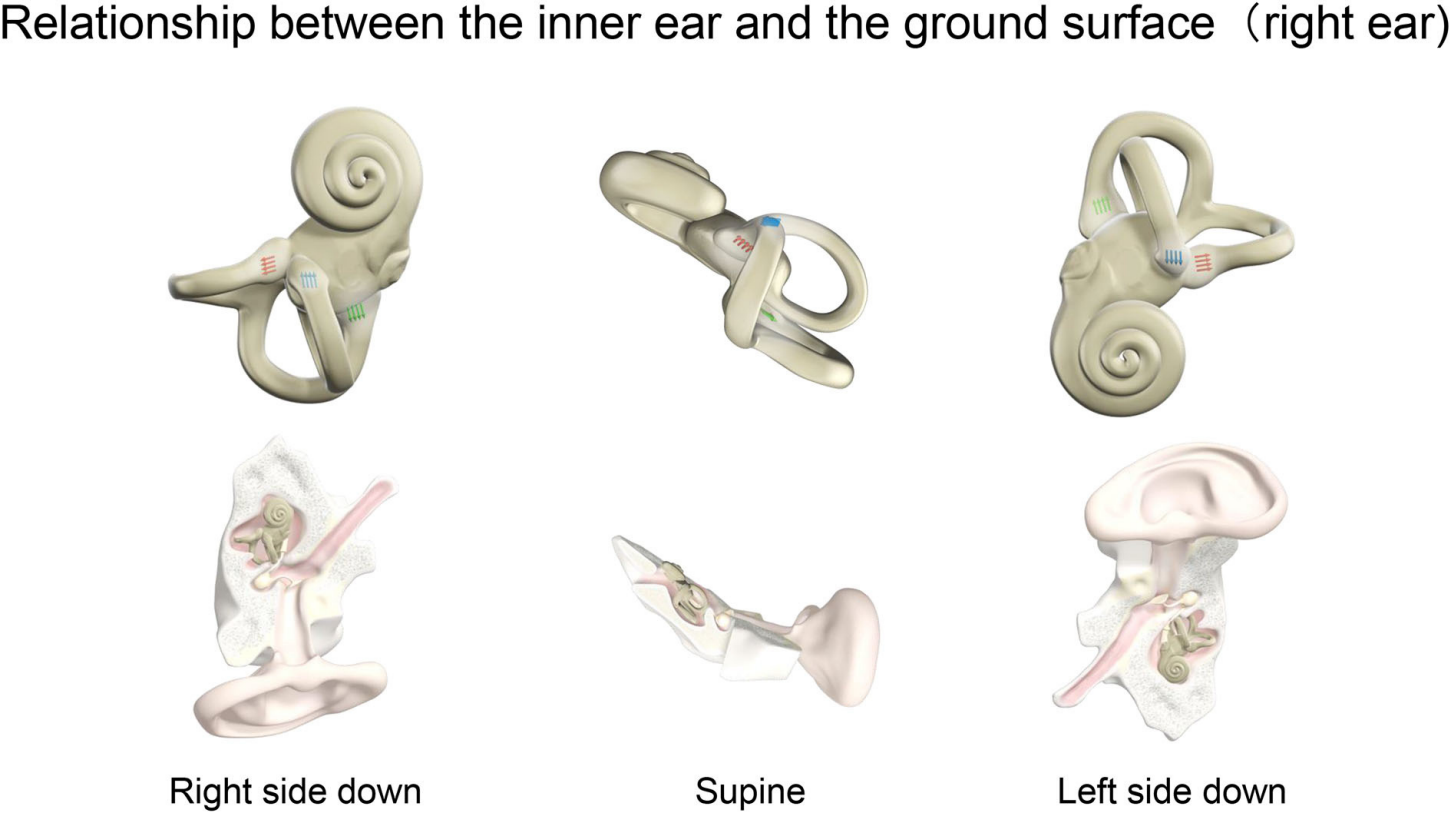
Relationship between the inner ear and the ground surface (right ear). The positional relationship of the inner ear for right ear side down, supine, and left ear side down in a person lying on the MRI bed. The right inner ear is viewed in the horizontal plane. The ampulla is shown for the three semicircular canals. Blue, red, and green arrows indicate the ampulla in the horizontal, anterior, and posterior semicircular canal, respectively. The direction of the arrows shows the stimulus direction of endolymph movement. In the horizontal canal, ampullopetal flow toward the ampulla causes a stronger stimulus. By contrast, ampullofugal flow leaving the ampulla causes a stronger stimulus in the anterior and posterior canals. Nystagmus, a fast component of eye movement, moves in the opposite direction to the arrows of the three semicircular canals combined.

In the male patient with endolymphatic hydrops in the right cochlea, ^17^O-labeled saline was administered intratympanically to the right ear. This patient also experienced vertigo symptoms and nystagmus, and video oculography (VOG) showed horizontal rotatory direction-changing positional geotropic nystagmus (Figure 4). His vertigo lasted for 6.5 h and was followed by several days of dizziness and nystagmus. In the horizontal component, the rapid phase of nystagmus in the left lower head position (injection side up) was leftward, and the frequency was low. By contrast, in the right lower head position (injection side down), the rapid phase changed to the rightward and increased frequency. Although the waveform in the supine position could not be obtained because of errors at 270 min after injection, we confirmed that the leftward nystagmus in the supine position changed to the rightward direction after 390 min in the later scan. The torsional component was clockwise in the right lower head position and counterclockwise in the left head lower position. The vertical component was always upward. The nystagmus showed no latency and no fatigue phenomenon. The amplitude and frequency of the nystagmus decreased gradually with time. The position of the vestibule with the head position is shown in Figure 5.

### MRI findings after ^17^O-labeled water injection into the inner ear

**Figure 6:**
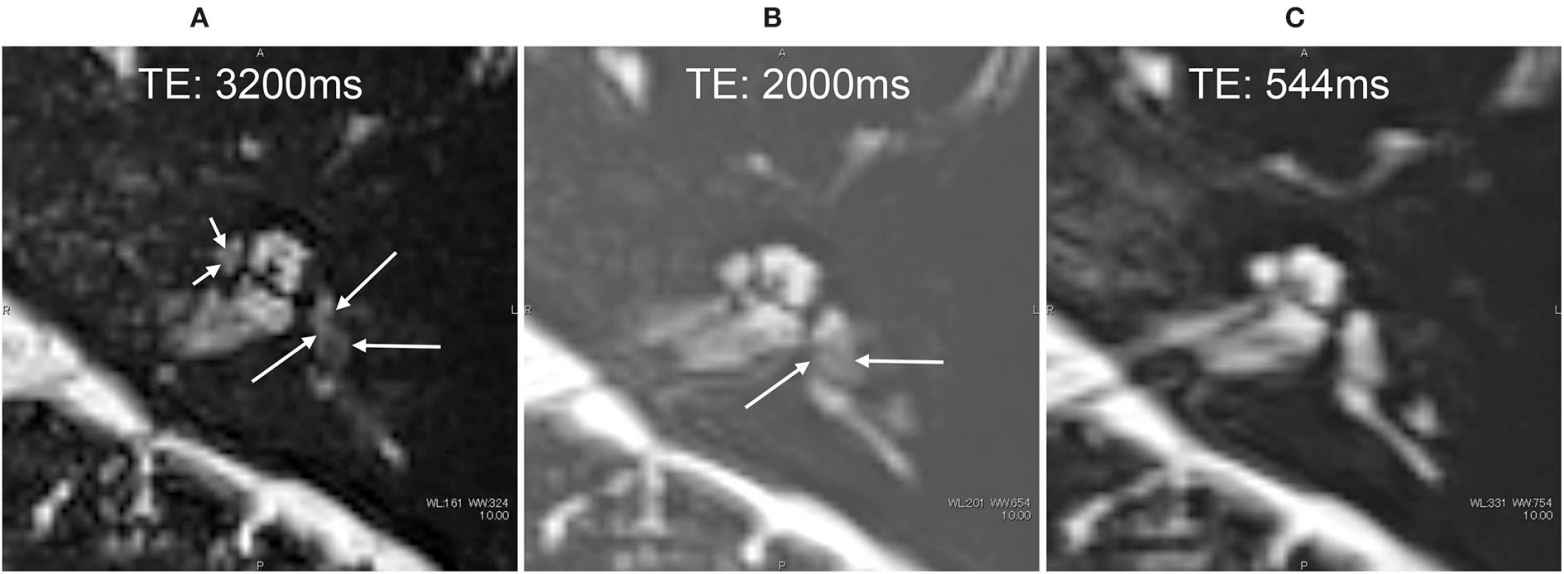
MR cisternography of the left ear in a 34-year-old male volunteer at the start of the scan. The MR scan was initiated 30 min after the intratympanic administration of ^17^O-labeled saline **(A–C)**. Images were obtained serially in the order of **(A**–**C)**. In the image with an echo time (TE) of 3,200 ms **(A)**, the signal of the whole vestibule (arrows) decreased because of the highest sensitivity to the T2-shortening effect of ^17^O-labeled saline. The anterior part of the cochlea also showed a decreased signal (short arrows). In the image with a TE of 2,000 ms **(B)**, the anatomy of the labyrinth and the contrast between the area with rich ^17^O-labeled water distribution (arrows) and that with poor ^17^O-labeled water distribution can be seen. In the image with a TE of 544 ms **(C)**, the T2-shortening effect of ^17^O-labeled water was almost unrecognizable, and the anatomy of the labyrinth could be seen.

**Figure 7:**
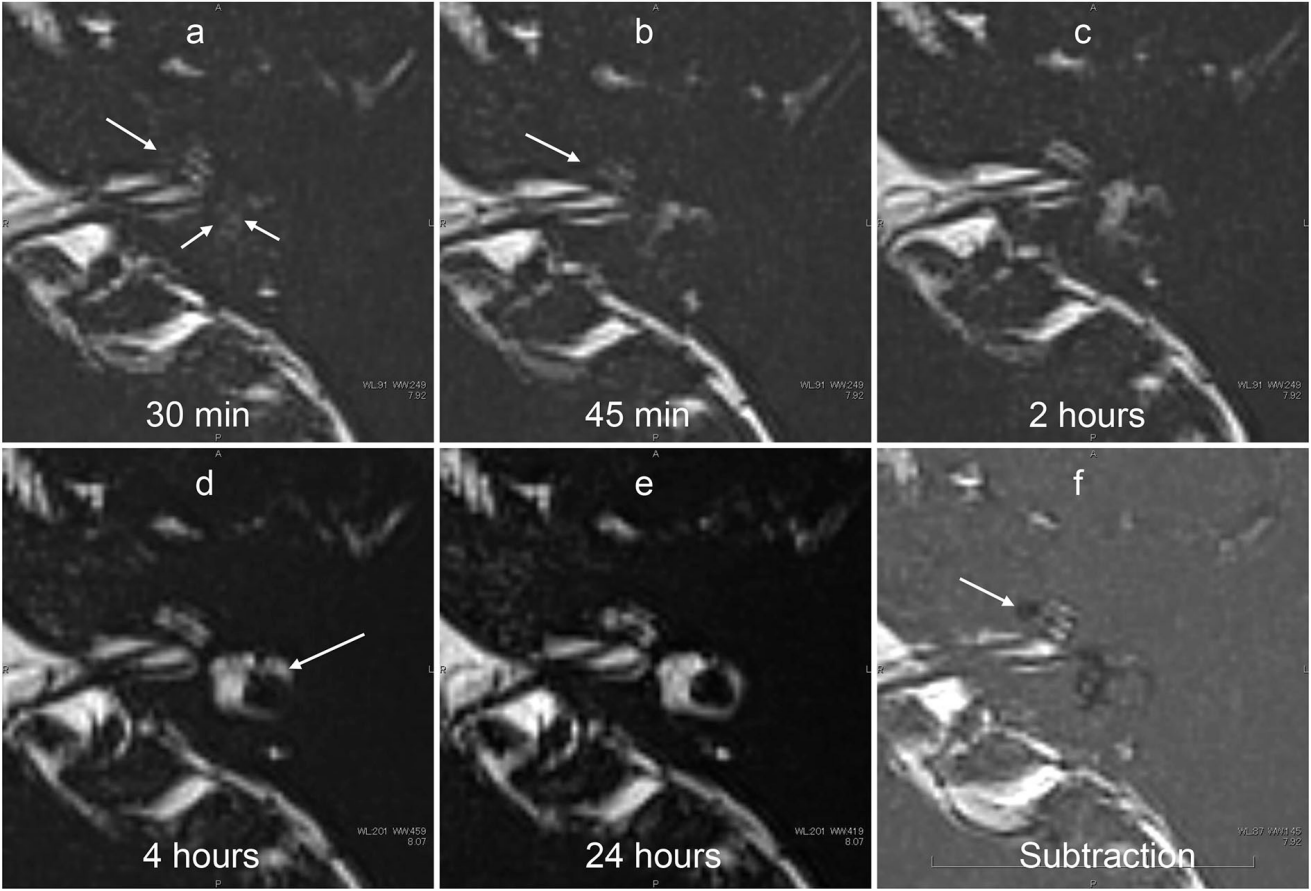
Serial MR cisternography (TE: 3,200 ms) of the left ear in a 34-year-old male volunteer (different volunteer from that shown in Figure 3) at 30 min **(a)**, 45 min **(b)**, 2 h **(c)**, 4 h **(d)**, and 24 h **(e)** after intratympanic administration of ^17^O-labeled saline. The subtraction image is also shown **(f)**. This subtraction image was generated as the value for **(b)** minus the value for a TE of 540 s × 0.18. The MR scan was initiated ~30 min after the intratympanic administration of ^17^O-labeled saline to the left middle ear. The images at the level of the cochlear nerve are shown. The signal decrease in the vestibule (short arrows in **a**) and in the anterior part of the basal cochlear turn (arrows in **a,b**) was most prominent in the image obtained at 30 min. At 45 min **(c)**, some parts of the cochlear (arrow) and the vestibular signal began to recover. At 2 h, the vestibular signal and the signal in the basal turn of the cochlea recovered further **(c)**. At 4 h, the signal for the lateral semicircular canal had recovered (arrows in **d**). At 24 h **(e)**, the signal for the left labyrinthine fluid had recovered to a level similar to that for the contralateral side (not shown). In the subtraction image **(f)**, an area with a rich distribution of ^17^O-labeled water was seen as a lower signal area (arrow). This subtraction image provided an overview of the distribution of ^17^O-labeled water and the anatomy of the labyrinth simultaneously in a single image.

**Figure 8:**
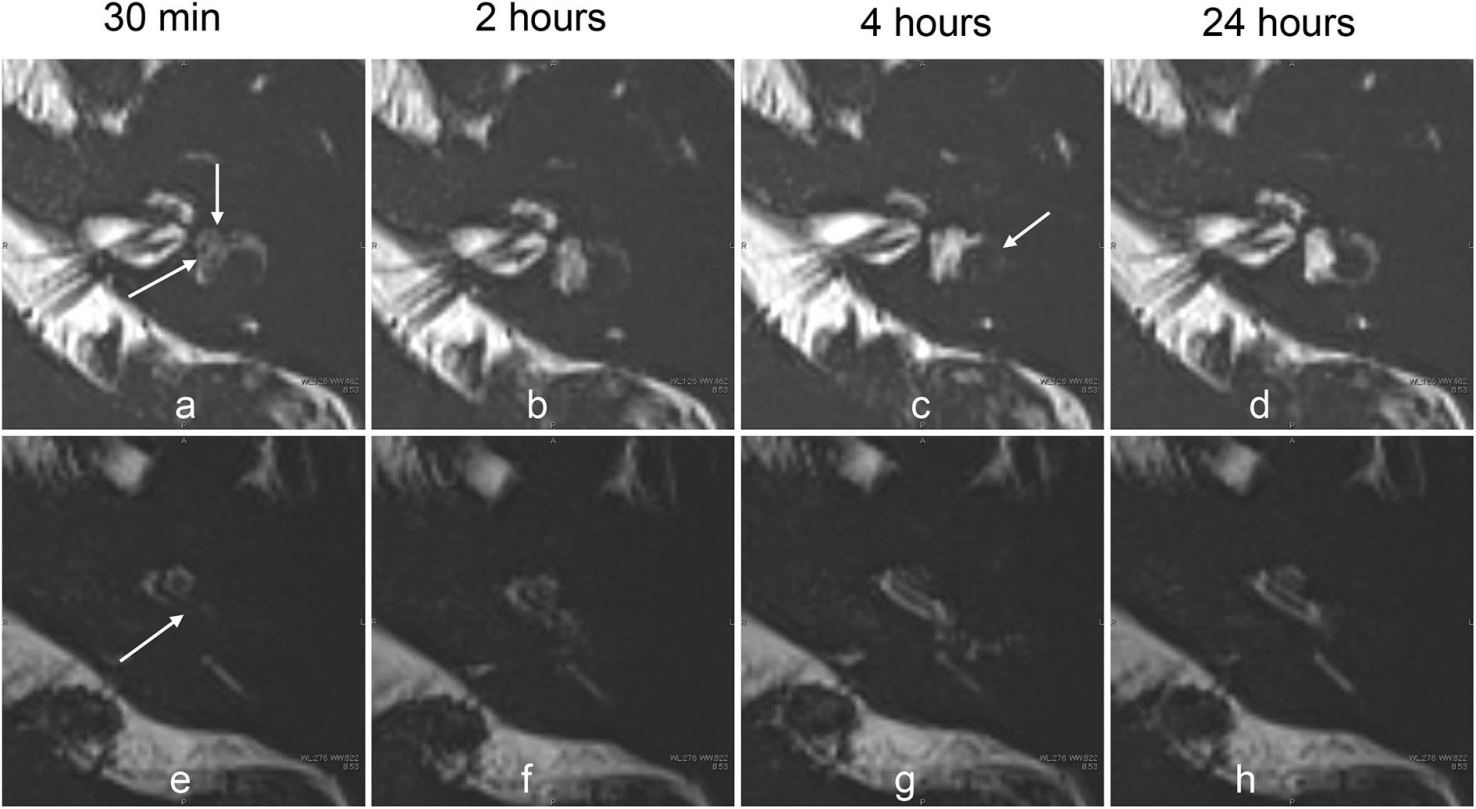
Serial MR cisternography (TE: 3,200 ms) of the left ear in a 33-year-old female volunteer at 30 min, and 2, 4, and 24 h after the intratympanic administration of ^17^O-labeled saline **(a–c)**. The MR scan was initiated ~30 min after the intratympanic administration of ^17^O-labeled saline into the left middle ear. The upper row **(a–d)** shows the images at the level of the cochlear nerve. The lower row **(e–h)** shows the images at the level of the cochlear aqueduct. The signal decreases in the vestibule (arrows, **a**) and in the basal turn of the cochlea (arrows, **e**) were most prominent in the image obtained at 30 min. The extent of the signal decrease in the vestibule in this volunteer is less than that for the volunteer whose images are shown in Figure 4. At 2 h, the vestibular signal and the signal in the basal turn of the cochlea began to recover **(b,f)**. At 4 h, the signal for the lateral semicircular canal decreased (arrows, **c**), and the signal for the cochlear basal turn recovered **(g)**. At 24 h **(d,h)**, the signal for the left labyrinthine fluid recovered to the level seen in the contralateral side (not shown).

**Figure 9:**
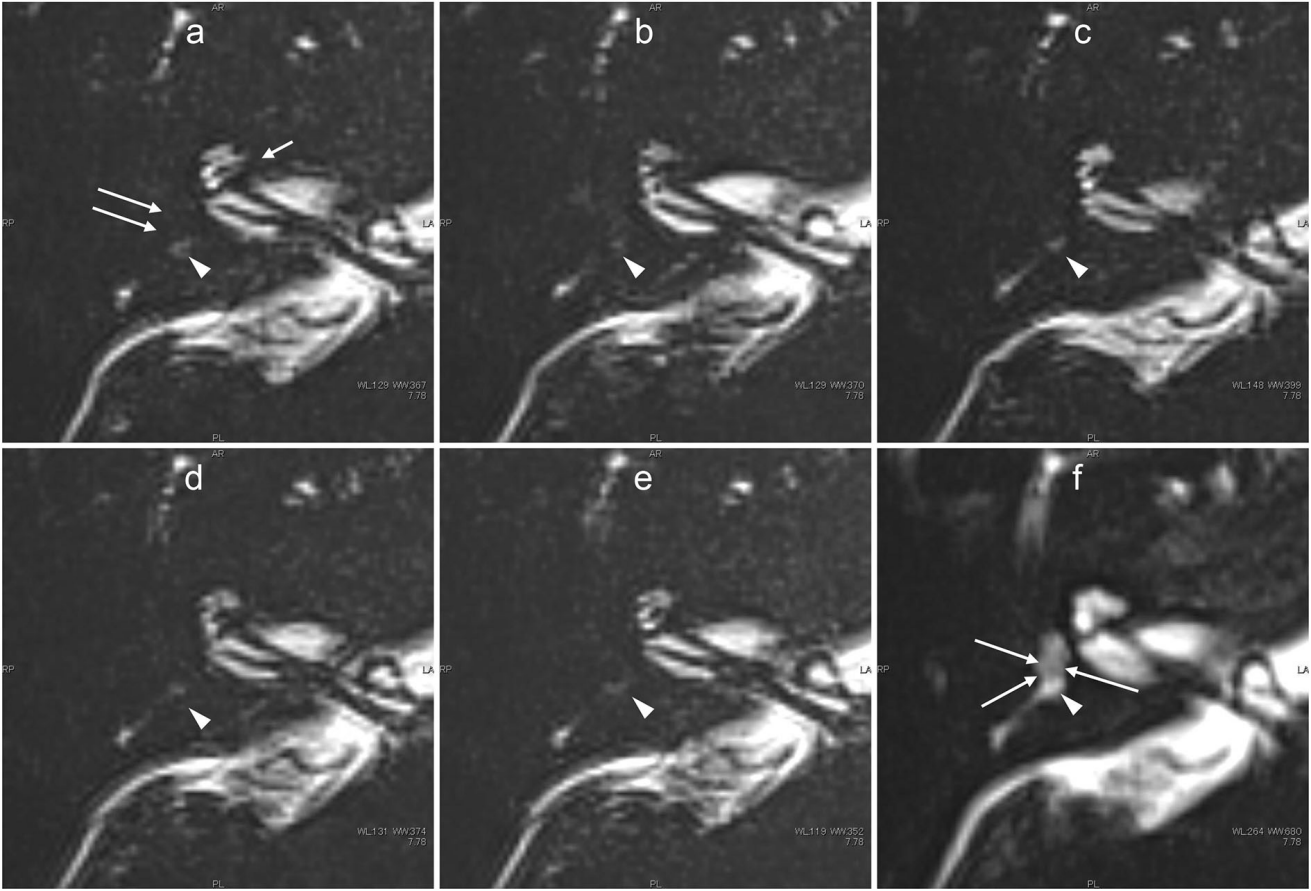
Images obtained from the male patient aged in his 40's with a history of acute low-tone sensorineural hearing loss 3 years previously but no history of vertigo who was currently asymptomatic. 3 years earlier, the patient had been shown to have cochlear endolymphatic hydrops by previous contrast-enhanced MR imaging using an intravenous gadolinium-based contrast agent. In this patient, five ultra-heavily T2-weighted images (6 min scan with a TE of 3,200 ms) were obtained serially **(a–e)**, and a 3 min scan image with a TE of 540 ms was obtained as a less sensitive scan to delineate the labyrinthine anatomy **(f)**. From the initial scan obtained ~30 min after intratympanic administration of ^17^O-labeled water **(a)**, the signals for the vestibular signal (arrows) and anterior part of the basal turn in the cochlea (short arrow) were almost completely lost because of the distribution of ^17^O-labeled water. Note that most of the vestibule showed a slightly decreased signal even in the less sensitive image with a TE of 540 ms (arrows, **f**). The concentration of intralabyrinthine ^17^O-labeled water was higher in this patient than in the volunteers. The signal for the posterior ampulla (arrowheads in **a**–**f**) decreased gradually from the initial phase **(a)** to the third phase **(c)**. From the third phase **(c)** to the fifth phase **(e)**, almost no signal was observed in the posterior ampulla.

In all participants, ^17^O-labeled water was found to be distributed in the inner ear on the injected side (Figures 6–9). That is, there was a decrease in signal in the inner ear on the injected side on T2-weighted MR images. In the image with an echo time (TE) of 3,200 ms, the whole vestibular signal decreased because of the high sensitivity to the T2-shortening effect of ^17^O-labeled saline (Figure 6A). The basal turn and anterior part of the second turn of the cochlea also showed decreased signals in the scala tympani, scala vestibuli, and scala media. The image for a TE of 2,000 ms (Figure 6B) showed the anatomy of the labyrinth and the contrast between the areas with rich and poor ^17^O-labeled water distribution. The image for a TE of 540 ms (Figure 6C) showed a nearly unrecognizable T2-shortening effect after administration of ^17^O-labeled water.

The images with shorter TE provided details on the anatomy of the labyrinth. Serially acquired images provided information about the time course of the spatial distribution of ^17^O-labeled water (Figures 7, 8). The separation between the endolymph and perilymph was not distinct in the cochlea and vestibule (Figures 6–9), and we could not identify the shape of the saccule and utricle. The distribution of ^17^O-labeled water to the posterior ampulla could be visualized in the longest TE images (Figures 6, 7, 9). In the patient, repeated scans for the longest TE images showed that ^17^O-labeled water distributed gradually to the posterior ampulla (Figure 9).

### Time course of the signal–intensity ratio

**Figure 10:**
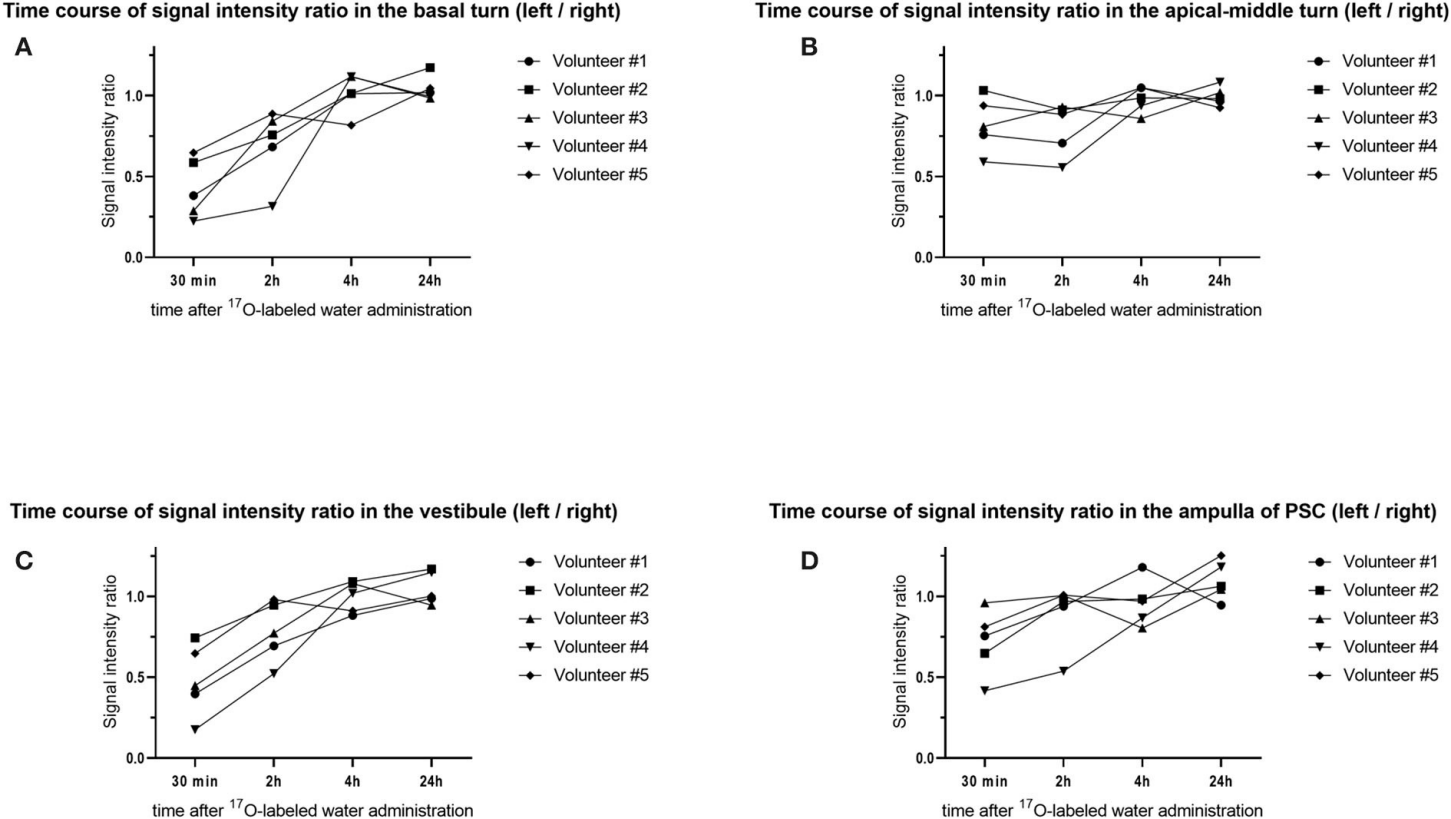
Time course of the signal–intensity ratio (SIR) in the five volunteers. The vertical axis shows the SIR and the horizontal axis shows the time elapsed since injection of the ^17^O-labeled water. Using the signal value for the right side as a control, each SIR was calculated as the signal intensity of the region of interest/signal intensity of the right ear. Time course of the SIR in **(A)** the basal turn of the cochlea, **(B)** the apical–middle turn of the cochlea, **(C)** the vestibule, and **(D)** the ampulla of the posterior semicircular canal (PSC). Note that the distribution of ^17^O-labeled water in the basal turn of the cochlea and vestibule at 30 min is obvious but is not obvious in the apical–middle turn. For the ampulla of the PSC, the distribution of ^17^O-labeled water at 30 min was seen in four volunteers.

The lowest SIR values were observed in the images obtained 30 min after the administration of ^17^O-labeled saline. In the volunteers, we obtained MR images at later times. After 2 h, the signal in the basal turn of the cochlea and the whole vestibular cavity had recovered slightly, and further signal recovery was observed after 4 h when the left–right difference disappeared almost completely. SIR values differed between the volunteers. At 30 min after intratympanic administration, the basal turn of the cochlea showed the lowest signal, followed by the vestibular space (Figure 10A). The apical–middle turn tended to have a slightly lower signal at 2 h than at 30 min, but this difference was not obvious (Figure 10B). The average SIR was higher for the ampulla of the posterior semicircular canal than that for the vestibular space but was lowest in both locations at 30 min after injection (Figures 10C,D). 24 h after injection, the SIR did not differ between the left and right sides of the inner ear at all measurement sites in all volunteers. Volunteers #1 and #4, whose dizziness persisted for more than 5 h, tended to have vestibular SIR values <0.4 at 30 min after the injection, and these values were lower than in the other volunteers. After 45 min from the injection of ^17^O-labeled saline, the patient with endolymphatic hydrops had severe vertigo symptoms, making it challenging to perform imaging. The SIR at 30 min after intratympanic administration of ^17^O-labeled saline was 0.2 in the basal turn of the cochlea, 0.8 in the apical-middle turn, 0.1 in the vestibular space, and 0.6 in the ampulla of the posterior semicircular canal, which was comparable to those in volunteers where the signal decrease was most evident.

## Discussion

This report is the first study in humans to show that intratympanic administration of ^17^O-labeled saline can produce contrast that can be examined using proton MRI of the inner ear. Intratympanic or intravenous GBCA administration is used as a contrast method for the separate visualization of endolymph and perilymph in the inner ear (10, 13, 19). Intratympanically administered GBCA distributes mainly into the perilymphatic fluid space and not into the endolymphatic space (11, 14). In the presence of endolymphatic hydrops, the endolymphatic space is seen as an enlarged low-signal area separated from the high-signal area of the perilymphatic space with GBCA distribution. However, GBCA is not suitable for use in patients with asthma, allergies, or renal insufficiency. If endolymphatic hydrops can be identified without the use of conventional gadolinium contrast agents, ^17^O-labeled saline enhancement allows examining patients with asthma, renal dysfunction, and allergy to contrast agents.

^17^O-labeled water has an indirect effect by shortening the T2 value in proton MRI. The R_2_ value is estimated as 3.33 s^−1^ 8. To detect low-concentration ^17^O-labeled water, a T2-weighted pulse sequence is required. In a previous study, a steady-state sequence was used in the evaluation of the brain parenchyma3. In the present study, we focused on the signal change for the inner ear lymph fluid, which has a very long T2 value. To detect the subtle shortening of T2 for the fluid with a high spatial resolution in a reasonable scan time, we used MR cisternography and a 3-D-turbo spin–echo sequence with an extremely long TE (3,200 ms). This kind of ultra-heavily T2-weighted (hT2W) imaging has not been used in clinical settings.

Systemic administration of 20% ^17^O-labeled water at a dose of 1 mL/kg caused no side effects in 14 participants in one study (3). Intrathecal administration of 10 mL of 10% ^17^O-labeled water caused no adverse effects in four patients in another study (8). ^17^O-labeled water is a safe but expensive tracer (several hundred US dollars per mL), and the routine use of large amounts of this tracer is not practical. Local administration of a small amount of this tracer might be practical, although the transitional positional vertigo following the intratympanic administration of this tracer should be explained in advance, and patients should be monitored closely. One has to explore the threshold level of the ^17^O-labeled saline concentration that does not produce vertigo. We need to set up an imaging protocol to identify low concentrations of ^17^O-labeled saline.

Analysis of the time course of the SIR showed that the lowest values, which represent the highest concentration of ^17^O-labeled water in the inner ear, occurred 30 min after intratympanic administration. We waited 30 min after intratympanic administration of ^17^O-labeled saline before the start of the initial MRI scan in the present study. The ^17^O-labeled saline penetrated both the perilymph and the endolymph in the cochlea and the vestibule. In the patient who underwent serial scans with a TE 3,200 ms, we could see the gradual filling of the posterior ampulla by ^17^O-labeled saline. In the present study, we have visually shown that the water in the perilymph reaches the endolymph in a short time. In addition, vertigo was prolonged in cases with a good distribution of ^17^O-labeled water and significant MRI signal reduction. These are the major discoveries of this study that have not been reported. The brain's delayed water dynamics or impaired glymphatic function is now intensively investigated as the cause of neurodegenerative diseases, including Alzheimer's disease and idiopathic normal pressure hydrocephalus (20–26). Impaired glymphatic system function (i.e., impaired waste clearance function) results in the deposition of amyloid beta and tau protein in the brain of patients with Alzheimer's disease. The ocular glymphatic system is also a hot topic in ophthalmology for the pathogenesis of neurodegeneration in glaucoma (20–24). The similarity of amyloid beta and tau protein deposition between Alzheimer's disease and glaucoma has been reported. The link between these diseases is also suggested based on the same glymphatic background (27, 28). Further research of the water dynamics using ^17^O-labeled water in the inner ear might open the door to reveal the mystery of the neurodegeneration in Ménière's disease.

The timing of 30 min after intratympanic administration of ^17^O-labeled saline was not optimal for discriminating endolymph from perilymph. Had we obtained much earlier images after intratympanic administration, we might have been able to visualize the signal difference between the endolymph and perilymph in the cochlea and vestibule. Obtaining MR images at earlier times would require intratympanic administration with the patient or participant already on the MR scanner table.

An animal study of the permeability of the endolymph–perilymph barrier using tritiated water reported that the permeability is 130 times higher for water than for K^+^ (29). A study in rats using tracers reported the following findings: a rapid turnover of water in the endolymph, perilymph, and cerebrospinal fluid because ^3^H_2_O equilibrated with plasma within a few min; slow entry of ^42^K and ^36^Cl into the perilymph because ^36^Cl equilibrated with the plasma after 2 h and ^42^K did not at 6 h; and an extremely slow entry of ^42^K and ^36^Cl into the endolymph because no equilibrium with plasma was obtained within the 5 h of the experiments (30).

Although evaluation of water permeability through the human endolymph–perilymph barrier has not been reported, this permeability is considered to be very high, as reported in animal experiments. In our study, the earliest sites of the distribution of ^17^O-labeled water were in the basal turn of the cochlea and the vestibular space, as also seen with intratympanic GBCA administration. This probably reflects the existence of a pathway for water administered into the tympanic cavity to enter the perilymph through the round window and the periphery of the stapes. However, 2 h after the injection, the signal tended to recover, which suggests that the decrease in concentration reflected the production and absorption of perilymph and endolymph or the diffusion of ^17^O-labeled water.

It is well-known that positional vertigo is caused by endolymphatic flow in the semicircular canal. In clinical settings, the procedure of tympanic space lavage with saline does not induce prolonged dizziness. No adverse effects of dizziness were reported in a case–control study involving intratympanic administration of saline (31). ^17^O-labeled water diffused into the inner ear may have caused endolymphatic flow in the semicircular canal with a change in the head position. Money and Myles examined the response to ingestion of 100–200 g deuterium oxide, which caused vigorous lateral positional nystagmus lasting some hours in humans accompanied by a directional characteristic opposite to that of postural alcohol nystagmus (32).

Deuterium oxide (“heavy water”) has a molecular weight of 20.030, as compared with 18.016 for water, and is thought to diffuse earlier into the cupula than the endolymph. If a sufficiently large specific gravity differential is maintained between the two, the cupula may act inappropriately as a gravity transducer. Koizuka et al. (33) demonstrated that both ethyl alcohol and heavy water affect the long-time constant of the vestibulo-ocular reflex and apogeotropic-type positional nystagmus lasted ~4 h in rabbits. These findings suggest that ethyl alcohol and heavy water directly alter the dynamics of the cupula–endolymph system in the vestibular end organs. The semicircular canal may be responsible for ethyl alcohol- or heavy water-induced positional nystagmus.

In the present study, the duration of vertigo was several hours, which was similar to that reported in previous animal studies using heavy water (32, 33). The VOG results showed no latency or attenuation in the nystagmus, suggesting a so-called light cupula (heavy endolymph) rather than canalolithiasis (34). The water component of the endolymph around the cupula is replaced by ^17^O-labeled water, which may make the cupula lighter. In the current study, increased endolymph density in the vestibule induced a light cupula phenomenon in the lateral and posterior semicircular canal according to the anatomical and gravitational situations and elicited persistent irritative nystagmus. Clinical light cupula syndrome was first proposed in 2004 (35), and various reports have been identified in the literature. In the present study, in which ^17^O-labeled water, a type of heavy water, was administered intratympanically, it can serve as a reproducible model of positional nystagmus in clinical light cupula syndrome.

The formula to convert the signal intensity to of ^17^O concentration has been reported (8). The concentration of ^17^O-labeled water can be estimated from the echo time (in s), signal intensity before and after administration, natural abundance ratio of ^17^O (0.038%), and R_2_ of ^17^O 3.33 s^−1^. Using MRI, we estimated that the concentration of ^17^O-labeled water administered intratympanically was diluted by 32–64 times in the vestibule in the patient whose concentration of ^17^O-labeled water was the highest of all the participants in the present study. The molecular weight of ^17^O-labeled water is 19 compared with 18 for normal water, and the specific gravity is slightly higher for ^17^O-labeled water. Therefore, the increase in the water density in the vestibule may have been 0.08–0.16% in this study. This percentage increase or decrease in the water density is caused by a 2–4°C decrease or increase in body temperature, respectively. The protein concentration in the endolymph is 20–30 mg/dL in healthy people and 182–348 mg/dL in people with Ménière's disease (36). Therefore, an increase in the endolymphatic protein concentration of 80–160 mg/dL corresponds to a 0.08–0.16% elevation in endolymph density. Our results suggest that the change in endolymph density can elicit caloric nystagmus and vertigo in people with Ménière's disease (i.e., the buoyancy theory).

Our study has some limitations. The number of participants was small because of the vertigo experienced after intratympanic administration of ^17^O-labeled water. This made statistical analysis difficult. Another limitation is individual variation in the round window permeability. A study of intratympanic GBCA administration found that round window permeability was absent in 5% of ears and poor in 13% of ears (37). In this study, the concentration of ^17^O-labeled water in the inner ear, or SIR, varied between volunteers. The distribution of ^17^O-labeled water to the inner ear was very rapid. It was clear that the contrast findings of the inner ear changed within a small timeframe after intratympanic injection. The temporal resolution can be improved by focusing on obtaining single types of echo time images (i.e., 3,200 ms), as we did in the patient.

Another limitation is that we did not perform a control study involving intratympanic administration of normal saline (^16^O-labeled saline) to confirm that the symptoms shown in the present study were caused by ^17^O-labeled saline. We also did not perform a contrast-enhanced study using GBCA in volunteers to visualize the individual anatomy of endolymphatic space.

## Conclusion

The inner ear water dynamics *in vivo* have not been clarified until now, and this study has revealed a part of it. In the future, elucidating the water dynamics in patients with endolymphatic hydrops, such as those with Ménière's disease, may help to elucidate the mechanism responsible for endolymphatic hydrops formation and vertigo attacks.

## Data availability statement

The original contributions presented in the study are included in the article/supplementary material, further inquiries can be directed to the corresponding author/s.

## Ethics statement

The studies involving human participants were reviewed and approved by the Nagoya University Clinical Research Review Board, Nagoya, Japan. The patients/participants provided their written informed consent to participate in this study.

## Author contributions

TY and SN designed the study. SN, MS, and TN coordinated and directed the project. TY, MK, and SS collected the clinical and imaging data. TY and SN wrote the report. SN, MS, and TN provided scientific direction. SN performed the image analysis. TY, SN, and KN performed the data analysis. All authors contributed to the article and approved the submitted version.

## Funding

This research was partially supported by Nagoya University Hospital Funding for Clinical Development.

## Conflict of interest

The authors declare that the research was conducted in the absence of any commercial or financial relationships that could be construed as a potential conflict of interest.

## Publisher's note

All claims expressed in this article are solely those of the authors and do not necessarily represent those of their affiliated organizations, or those of the publisher, the editors and the reviewers. Any product that may be evaluated in this article, or claim that may be made by its manufacturer, is not guaranteed or endorsed by the publisher.

## References

[1] Zhu XH Zhang N Zhang Y Zhang X Ugurbil K Chen W. In vivo 17O NMR approaches for brain study at high field. NMR Biomed. (2005) 18:83–103. 10.1002/nbm.930 15770611

[2] Kudo K Harada T Kameda H Uwano I Yamashita F Higuchi S. Indirect MRI of (17) o-labeled water using steady-state sequences: Signal simulation and preclinical experiment. J Magn Reson Imag. (2018) 47:1373–9. 10.1002/jmri.25848 28861934

[3] Kudo K Harada T Kameda H Uwano I Yamashita F Higuchi S. Indirect proton MR imaging and kinetic analysis of 17O-labeled water tracer in the brain. Magn Reson Med Sci. (2018) 17:223–30. 10.2463/mrms.mp.2017-0094 29142152 PMC6039783

[4] Atkinson IC Thulborn KR. Feasibility of mapping the tissue mass corrected bioscale of cerebral metabolic rate of oxygen consumption using 17-oxygen and 23-sodium MR imaging in a human brain at 94 T. Neuroimage. (2010) 51:723–33. 10.1016/j.neuroimage.2010.02.056 20188194

[5] Cui W Zhu X-H Vollmers ML Colonna ET Adriany G Tramm B. Non-invasive measurement of cerebral oxygen metabolism in the mouse brain by ultra-high field (17)O MR spectroscopy. J Cereb Blood Flow Metab. (2013) 33:1846–9. 10.1038/jcbfm.2013.172 24064490 PMC3851910

[6] Hoffmann SH Radbruch A Bock M Semmler W Nagel AM. Direct (17)O MRI with partial volume correction: first experiences in a glioblastoma patient. MAGMA. (2014) 27:579–87. 10.1007/s10334-014-0441-8 24687775

[7] Hopkins AL Lust WD Haacke EM Wielopolski P Barr RG Bratton CB. The stability of proton T2 effects of oxygen-17 water in experimental cerebral ischemia. Magn Reson Med. (1991) 22:167–74. 10.1002/mrm.1910220118 1798391

[8] Sugimori H Kameda H Harada T Ishizaka K Kajiyama M Kimura T. Quantitative magnetic resonance imaging for evaluating of the cerebrospinal fluid kinetics with 17O-labeled water tracer: a preliminary report. Magn Reson Imaging. (2022) 87:77–85. 10.1016/j.mri.2021.12.005 34968701

[9] Leung K. “*17O-Labeled water.,” Molecular Imaging and Contrast Agent Database (MICAD)*. Bethesda, MD: National Center for Biotechnology Information (US) (2010). 20641179

[10] Nakashima T Naganawa S Sugiura M Teranishi M Sone M Hayashi H. Visualization of endolymphatic hydrops in patients with Meniere's disease. Laryngoscope. (2007) 117:415–20. 10.1097/MLG.0b013e31802c300c 17279053

[11] Pyykkö I Zou J Gürkov R Naganawa S Nakashima T. Imaging of temporal bone. Adv Otorhinolaryngol. (2019) 82:12–31. 10.1159/000490268 30947168

[12] Zou J Poe D Bjelke B Pyykko I. Visualization of inner ear disorders with MRI in vivo: from animal models to human application. Acta Otolaryngol Suppl. (2009) 5:22–31. 10.1080/00016480902729850 19221903

[13] Naganawa S Satake H Kawamura M Fukatsu H Sone M Nakashima T. Separate visualization of endolymphatic space, perilymphatic space and bone by a single pulse sequence; 3D-inversion recovery imaging utilizing real reconstruction after intratympanic Gd-DTPA administration at 3 Tesla. Eur Radiol. (2008) 18:920–4. 10.1007/s00330-008-0854-8 18324405

[14] Nakashima T Pyykkö I Arroll MA Casselbrant ML Foster CA Manzoor NF. Meniere's disease. Nat Rev Dis Primers. (2016) 2:16028. 10.1038/nrdp.2016.28 27170253

[15] Naganawa S Nakashima T. Visualization of endolymphatic hydrops with MR imaging in patients with Ménière's disease and related pathologies: current status of its methods and clinical significance. Jpn J Radiol. (2014) 32:191–204. 10.1007/s11604-014-0290-4 24500139

[16] Mugler JP III. Optimized three-dimensional fast-spin-echo MRI. J Magn Reson Imaging. (2014) 39:745–67. 10.1002/jmri.24542 24399498

[17] Morimoto K Yoshida T Kobayashi M Sugimoto S Nishio N Teranishi M. Significance of high signal intensity in the endolymphatic duct on magnetic resonance imaging in ears with otological disorders. Acta Otolaryngol. (2020) 140:818–22. 10.1080/00016489.2020.1781927 32646259

[18] Yoshida T Kobayashi M Sugimoto S Teranishi M Naganawa S Sone M. Evaluation of the blood-perilymph barrier in ears with endolymphatic hydrops. Acta Otolaryngol. (2021) 141:736–41. 10.1080/00016489.2021.1957500 34346271

[19] Gürkov R Flatz W Louza J Strupp M Ertl-Wagner B Krause E. Herniation of the membranous labyrinth into the horizontal semicircular canal is correlated with impaired caloric response in Ménière's disease. Otol Neurotol. (2012) 33:1375–9. 10.1097/MAO.0b013e318268d087 22918115

[20] Lohela TJ Lilius TO Nedergaard M. The glymphatic system: implications for drugs for central nervous system diseases. Nat Rev Drug Discov. (2022) 3:9 10.1038/s41573-022-00500-9 35948785

[21] Wostyn P. Do normal-tension and high-tension glaucoma result from brain and ocular glymphatic system disturbances, respectively? Eye. (2021) 35:2905–6. 10.1038/s41433-020-01219-w 33041336 PMC8452763

[22] Nakashima T Sone M Teranishi M Yoshida T Terasaki H Kondo M. perspective from magnetic resonance imaging findings of the inner ear: relationships among cerebrospinal, ocular and inner ear fluids. Auris Nasus Larynx. (2012) 39:345–55. 10.1016/j.anl.2011.05.005 21871749

[23] Taoka T Naganawa S. Imaging for central nervous system (CNS) interstitial fluidopathy: disorders with impaired interstitial fluid dynamics. Jpn J Radiol. (2021) 39:1–14. 10.1007/s11604-020-01017-0 32653987 PMC7813706

[24] Taoka T Naganawa S. Glymphatic imaging using MRI. J Magn Reson Imaging. (2020) 51:11–24. 10.1002/jmri.26892 31423710

[25] Rasmussen MK Mestre H Nedergaard M. The glymphatic pathway in neurological disorders. Lancet Neurol. (2018) 17:1016–24. 10.1016/S1474-4422(18)30318-1 30353860 PMC6261373

[26] Bae YJ Choi BS Kim J-M Choi J-H Cho SJ Kim JH. Altered glymphatic system in idiopathic normal pressure hydrocephalus. Parkinsonism Relat Disord. (2021) 82:56–60. 10.1016/j.parkreldis.2020.11.009 33248394

[27] Sen S Saxena R Tripathi M Vibha D Dhiman R. Neurodegeneration in Alzheimer's disease and glaucoma: overlaps and missing links. Eye. (2020) 34:1546–53. 10.1038/s41433-020-0836-x 32152519 PMC7608361

[28] Wostyn P De Groot V Van Dam D Audenaert K Killer HE De Deyn PP. Age-related macular degeneration, glaucoma and Alzheimer's disease: amyloidogenic diseases with the same glymphatic background? Cell Mol Life Sci. (2016) 73:4299–301. 10.1007/s00018-016-2348-1 27572287 PMC11108361

[29] Konishi T Hamrick PE Mori H. Water permeability of the endolymph-perilymph barrier in the guinea pig cochlea. Hear Res. (1984) 15:51–8. 10.1016/0378-5955(84)90224-7 6480523

[30] Sterkers O Saumon G Tran Ba Huy P Amiel CK. Cl, and H2O entry in endolymph, perilymph, and cerebrospinal fluid of the rat. Am J Physiol. (1982) 243:F173–80. 10.1152/ajprenal.1982.243.2.F173 7114216

[31] Araújo MFS Oliveira CA Bahmad FM Jr. Intratympanic dexamethasone injections as a treatment for severe, disabling tinnitus: does it work? Arch Otolaryngol Head Neck Surg. (2005) 131:113–7. 10.1001/archotol.131.2.113 15723941

[32] Money KE Myles WS. Heavy water nystagmus and effects of alcohol. Nature. (1974) 247:404–5. 10.1038/247404a0 4544739

[33] Koizuka I Takeda N Kubo T Matsunaga T Cha CI. Effects of ethyl alcohol and heavy-water administration on vestibulo-ocular reflex in rabbits. ORL J Otorhinolaryngol Relat Spec. (1989) 51:151–5. 10.1159/000276050 2734005

[34] Imai T Matsuda K Takeda N Uno A Kitahara T Horii A. Light cupula: the pathophysiological basis of persistent geotropic positional nystagmus. BMJ Open. (2015) 5:e006607. 10.1136/bmjopen-2014-006607 25586370 PMC4298092

[35] Hiruma K Numata T. Positional nystagmus showing neutral points. ORL J Otorhinolaryngol Relat Spec. (2004) 66:46–50. 10.1159/000077234 15107588

[36] Silverstein H Schuknecht HF. Biochemical studies of inner ear fluid in man. Changes in otosclerosis, Meniere's disease, and acoustic neuroma. Arch Otolaryngol. (1966) 84:395–402. 10.1001/archotol.1966.00760030397003 5921712

[37] Yoshioka M Naganawa S Sone M Nakata S Teranishi M Nakashima T. Individual differences in the permeability of the round window: evaluating the movement of intratympanic gadolinium into the inner ear. Otol Neurotol. (2009) 30:645–8. 10.1097/MAO.0b013e31819bda66 19415042

